# PReS-FINAL-2176: Declines in levels of disease activity and physical disability in children with juvenile idiopathic arthritis seen in standard clinical care over the last 25 years

**DOI:** 10.1186/1546-0096-11-S2-O11

**Published:** 2013-12-05

**Authors:** A Consolaro, S Lanni, F Minoia, S Davì, S Dalprà, B Schiappapietra, V Muratore, A Martini, A Ravelli

**Affiliations:** 1 Pediatria II, Istituto Giannina Gaslini, Genoa, Italy; 2 Pediatria, IRCCS Fondazione Policlinico San Matteo, Pavia, Italy; 3 University of Genoa, Genoa, Italy

## Introduction

Over the last 3 decades there have been important advances in the management of juvenile idiopathic arthritis (JIA), which include the introduction of methotrexate and, later on, the tendency toward its earlier initiation, the widespread use of intra-articular corticosteroid injections, and, more recently, the availability of the biologic response modifiers. Although this therapeutic progress is likely to have led to a marked improvement in the outlook of children with JIA, the prognostic impact of the newer therapeutic modalities is still poorly documented.

## Objectives

To evaluate the change in the measures of disease activty and phisical function in JIA over the last 25 years.

## Methods

The clinical information recorded during visits made in children with JIA from January 1987 to March 2012 was retrieved from the study center database. Visits were divided in the following time intervals: 1987-1995 (n = 826), 1996-2000 (n = 1,337), 2001-2005 (n = 2,022), 2006-2012 (n = 2,317). Measures of disease activity included the physician's and parent's global ratings (both made on a 0-10 cm visual analog scale, VAS), the parent's pain rating (made on a 0-10 cm VAS) and the count of joints with swelling, pain on motion/tenderness and active disease. Measures of disability included the count of joints with restricted motion and a physical function tool (the Childhood Health Assessment Questionnaire, CHAQ before March 2007 and the Juvenile Arthritis Functionality Scale, JAFS after that date). To enable comparability of functional ability evaluations, both CHAQ and JAFS scores were converted to a 0-10 scale (0 = best; 10 = worst). Parent's global and pain ratings as well as functional ability assessment were not available for visits made prior to 1995 because before this year these assessments were made on a scale not comparable with those used subsequently.

## Results

We observed a progressive decline in the levels of disease activity and physical disability over time among children with JIA seen from the mid of the 1980s to the 2010s. This finding confirms the notion that the recent advances in the management of JIA have led to a substantial improvement in disease prognosis.

**Table 1:** 

	Physician global (0-10)	Active joints	Swollen jointst	Tender joints	Restricted joints	Parent global (0-10)	Parent pain (0-10)	Physical function (0-10)
1988-1996	5.4 (2.8)	8.2 (9.4)	7.9 (8.3)	6.3 (8.0)	8.0 (10.4)	-	-	-
1996-2000	5.0 (3.4)	5.7 (8.4)	4.3 (5.8)	4.3 (7.9)	5.5 (9.5)	2.8 (2.5)	2.5 (2.5)	1.7 (2.0)
2001-2005	4.0 (3.5)	3.8 (5.7)	2.9 (4.2)	3.3 (6.0)	3.1 (6.2)	2.4 (2.5)	2.4 (2.7)	1.3 (1.8)
2006-2012	2 (2.7)	2.2 (4.5)	1.8 (3.8)	1.9 (4)	1.9 (4.2)	2.1 (2.5)	1.9 (2.6)	0.4 (0.9)

## Conclusion

We observed a progressive decline in the levels of disease activity and physical disability over time among children with JIA seen from the mid of the 1980s to the 2010s. This finding confirms the notion that the recent advances in the management of JIA have led to a substantial improvement in disease prognosis.

## Disclosure of interest

None declared.

